# Supplementary figures and images for: Disordered regions and folded modules in CAF-1 promote histone deposition in Schizosaccharomyces pombe
Source: eLife. 2024 Feb 20;12:RP91461. doi: 10.7554/eLife.91461 (PMC10942606; doi:10.7554/eLife.91461)

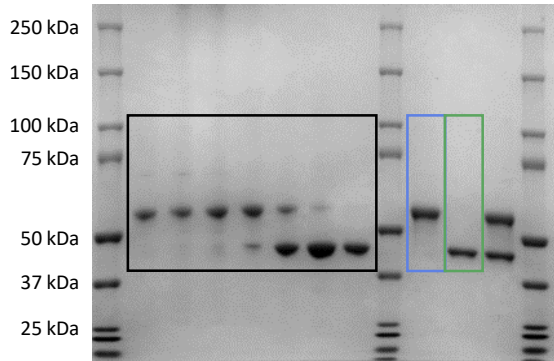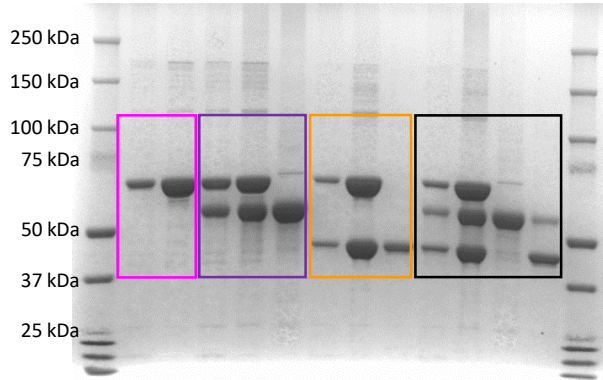

Supplement: Figure 1—figure supplement 2—source data 1. [file elife-91461-fig1-figsupp2-data1.pdf]

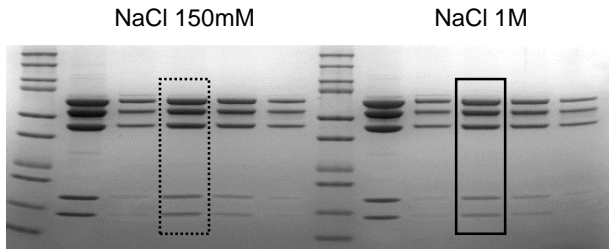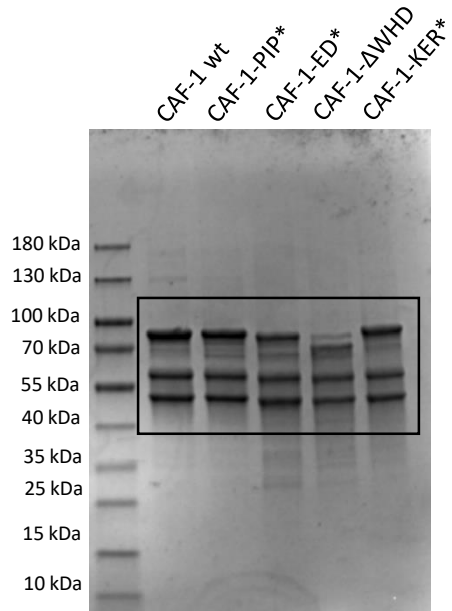

Supplement: Figure 2—source data 1. [file elife-91461-fig2-data1.pdf]

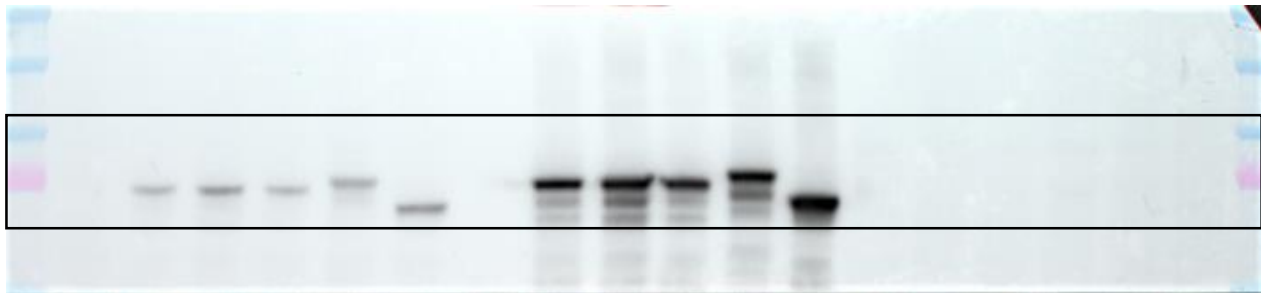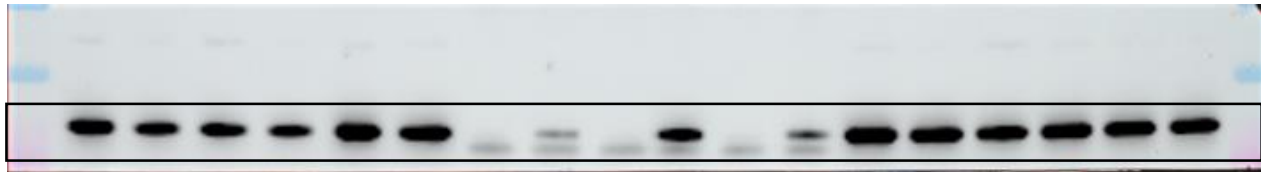

Supplement: Figure 6—source data 1. [file elife-91461-fig6-data1.pdf]
