# Supplementary material for: Disordered regions and folded modules in CAF-1 promote histone deposition in Schizosaccharomyces pombe
Source: eLife. 2024 Feb 20;12:RP91461. doi: 10.7554/eLife.91461 (PMC10942606; doi:10.7554/eLife.91461)

Figure 3A

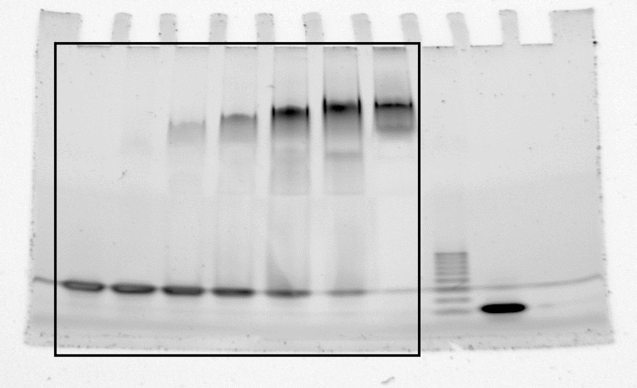

Figure 3D

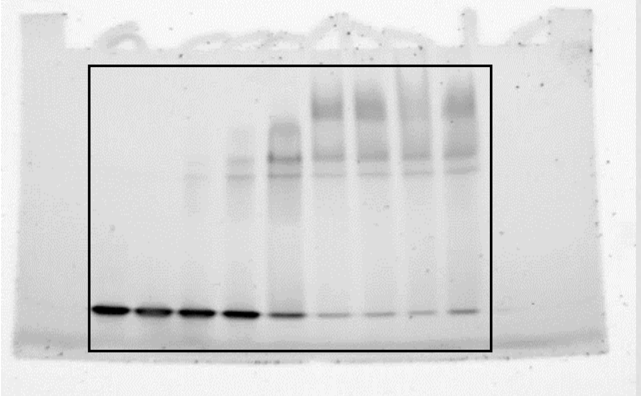

Figure 3G

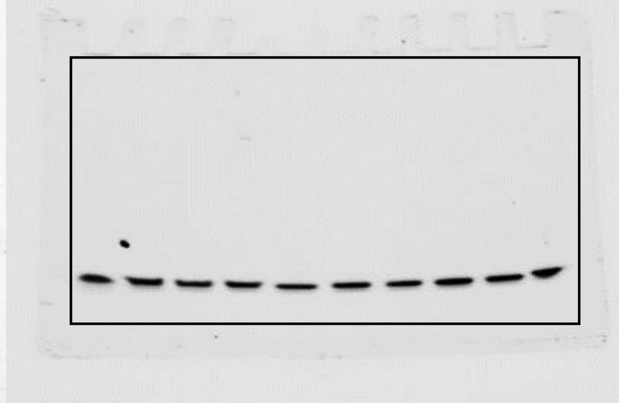

Figure 3 – figure supplement 1A

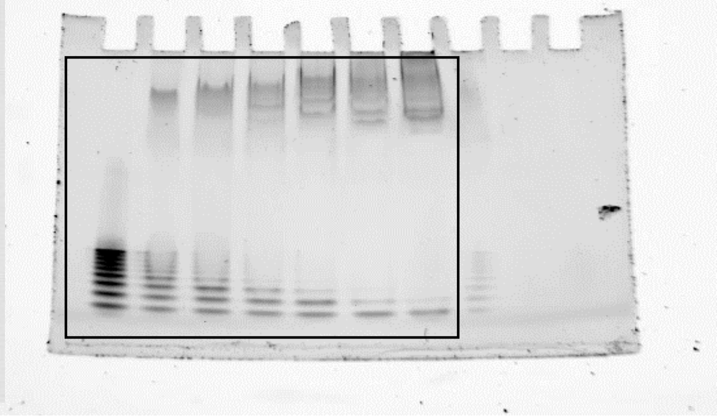

Figure 3 – figure supplement 2A

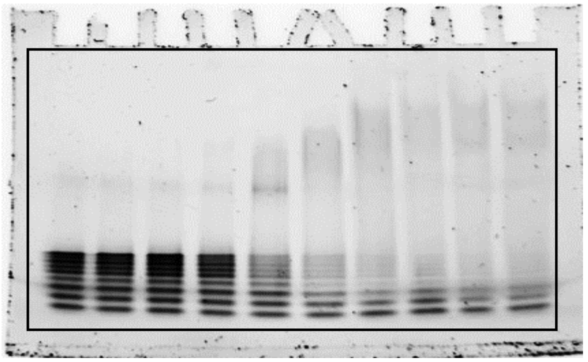

Figure 3 – figure supplement 2B

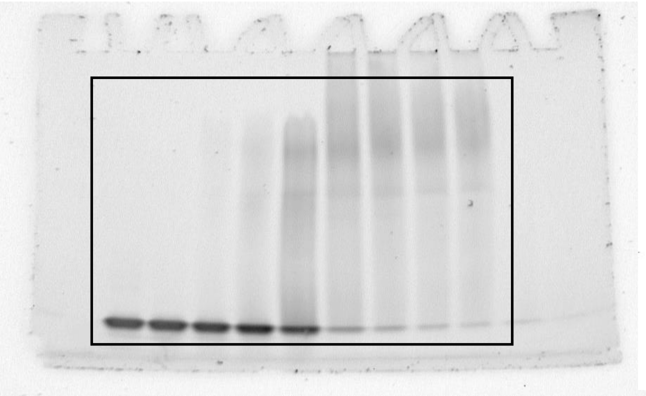

Figure 3 – figure supplement 2D

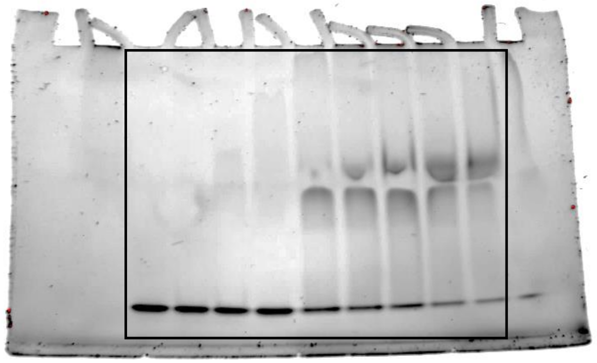

Figure 3 – figure supplement 2E

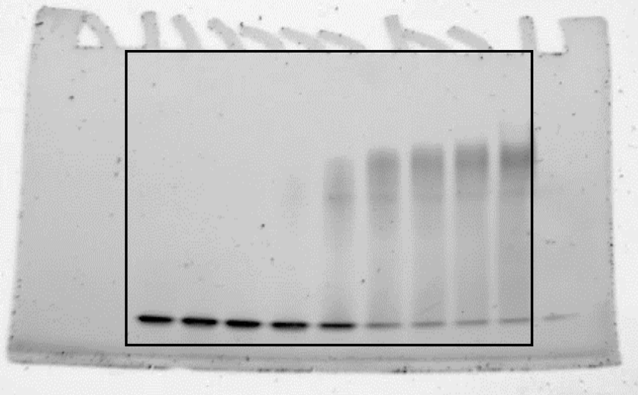

Figure 3 – figure supplement 2F

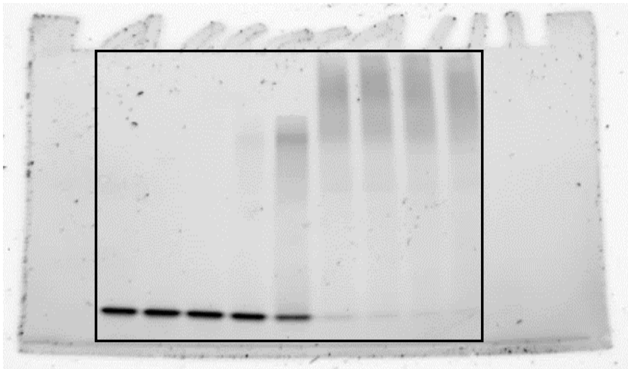

Figure 3 – figure supplement 2G (left upper panel)

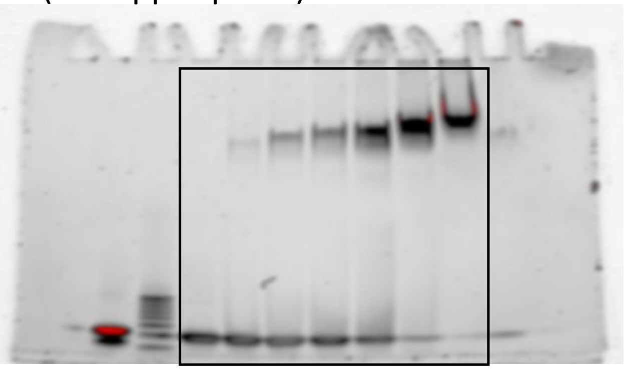

Figure 3 – figure supplement 2G (right upper panel)

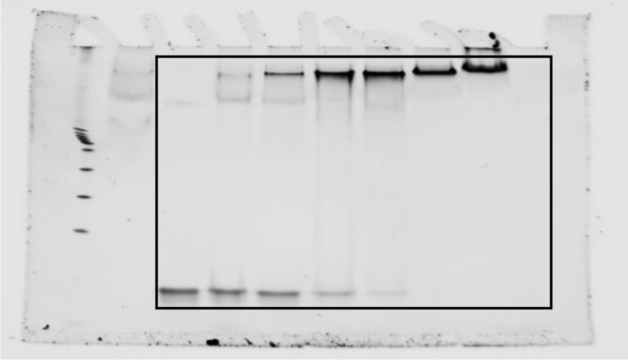

Figure 3 – figure supplement 2G (left lower panel)

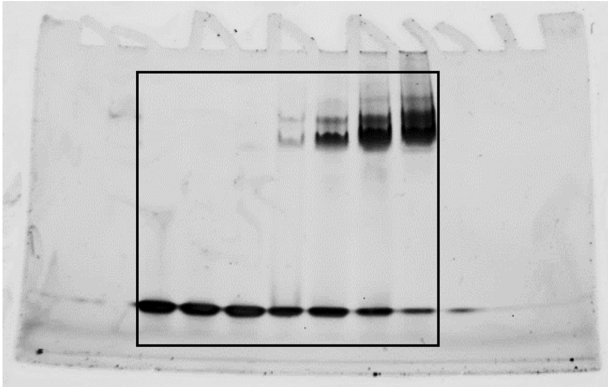

Figure 3 – figure supplement 2G (right lower panel)

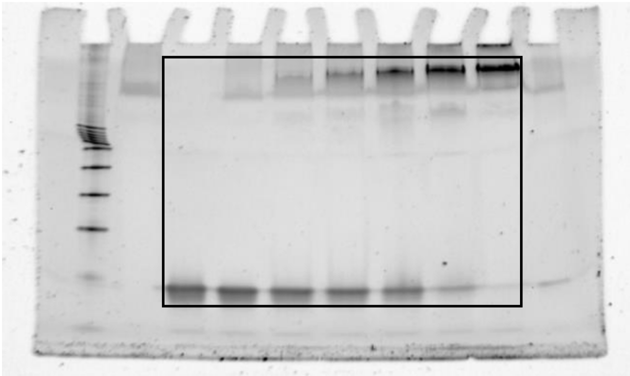

Figure 3 – figure supplement 4B

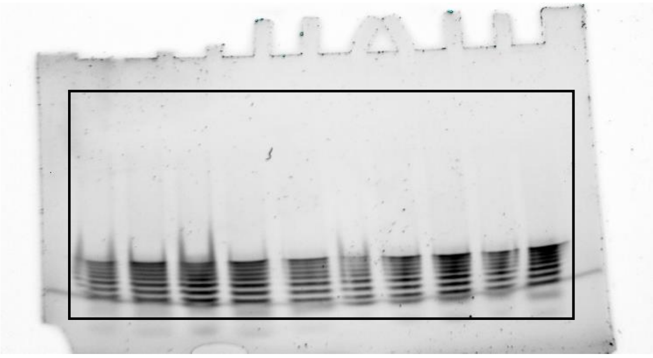

Supplement: Figure 3—source data 1. [file elife-91461-fig3-data1.pdf]
