# Supplementary material for: Disordered regions and folded modules in CAF-1 promote histone deposition in Schizosaccharomyces pombe
Source: eLife. 2024 Feb 20;12:RP91461. doi: 10.7554/eLife.91461 (PMC10942606; doi:10.7554/eLife.91461)
Supplement: Supplementary file 1. — (A) Experimental information and modelling of SAXS data. (B) Yeast strains used in this study. [file elife-91461-supp1.docx]

**Supplementary file 1a: Experimental information and modelling of SAXS data**

| **Sample details** | **Pcf1_KER** | ***Sp*CAF-1** | ***Sp*CAF-1+H3-H4** |
| --- | --- | --- | --- |
| Organism | *S. Pombe* | | |
| Source (catalogue No. or reference) | Recombinant proteins (See Methods) | | |
| UniProt sequence ID (residues in construct) | Q1MTN9 | Q1MTN9-O13985- Q9Y825 | Q1MTN9-O13985-Q9Y825-P09988-P09322 |
| Extinction coefficient [A_280_, 0.1%(*w/v*)] | 0.207 | 1.15 | 1.045 |
| M from chemical composition (Da) | 14 410 | 167 386 | 194 130 |
| SEC–SAXS column | S200 5/150 Increase | | |
| Loading concentration (mg ml^−1^) | 0.7 | 3.3 | 2.6 |
| Injection volume (µl) | 45 | 50 | 50 |
| Flow rate (ml min^−1^) | 0.3 | 0.3 | 0.3 |
| Solvent (solvent blanks taken from SEC flowthrough prior to elution of protein) | Tris 10 mM, NaCl 150 mM, β-mercaptoethanol 5 mM, pH 8 | | |
| **SAXS data-collection parameters.** |  |  |  |
| Instrument/data processing | BioSAXS on the SWING beamline at Synchrotron SOLEIL(Thureau et al. 2021) | | |
| Wavelength (Å) | 1.0332 | | |
| Beam size (µm) | 500x200 | | |
| Camera length (m) | 2.00 | | |
| q measurement range (Å^−1^); q = 4πsin(θ)/λ  (2θ: scattering angel & λ the x-ray wavelength) | 0.00410–0.5516 | | |
| Absolute scaling method | Comparison with scattering from 1 mm pure H_2_O | | |
| Normalization | To transmitted intensity by beam-stop counter | | |
| Monitoring for radiation damage | data frame-by-frame comparison | | |
| Exposure time | Continuous 1 s data-frame measurements of SEC elution | | |
| Sample configuration | SEC–SAXS with thermalized quartz capillary (ID 1.5mm) | | |
| Sample temperature (°C) | 20 | | |
| **Software employed for SAXS data reduction, analysis and interpretation** |  | | |
| SAXS data reduction | I(q) versus q, buffer subtraction & frames selection using Foxtrot 3.10 ^a^ | | |
| Extinction coefficient estimate | ProtParam(Wilkins et al. 1999) | | |
| Basic analyses: Guinier, *P(r)*, MW | PRIMUSqt from ATSAS 3.2.1(Manalastas-Cantos et al. 2021) | | |
| Atomic structure modelling | Dadimodo(Rudenko et al. 2019) (https://dadimodo.synchrotron-soleil.fr/) | | |
| Missing sequence modelling | MODELLER(Webb and Sali 2014) | | |
| Three-dimensional graphic model representations | PyMOL v.0.99 | | |
| **Structural parameters** |  |  |  |
| Guinier analysis |  |  |  |
| I(0) (cm^−1^) | 0.0184 ± 1E-4 | 0.0279 ± 1E-4 | 0.0241 ± 1E-4 |
| R_g_ (Å) | 44.75 ± 0.31 | 48.79 ± 0.29 | 59.02 ± 0.32 |
| q_min_ (Å^−1^) | 0.0055 | 0.0547 | 0.00593 |
| qR _g_ max (q min = 0.0066 Å^−1^) | 1.1 | 1.29 | 1.29 |
| Coefficient of correlation, R^2^ | 0.98 | 0.99 | 1 |
| M from Vc (ratio to predicted) | 12300 (0.85) | 168000 (1.00) | 196800 (1.1) |
| P(r) analysis |  |  |  |
| I(0) (cm^−1^) | 0.0186 ± 1E-5 | 0.0285 ± 1E-4 | 0.0248 ± 1E-4 |
| R_g_ (Å) | 48.43 ± 0.59 | 52.88 ± 0.76 | 65.37 ± 0.68 |
| d_max_ (Å) | 210 | 220 | 270 |
| q range (Å^−1^) | 0.0041 to 0.50 | 0.0055 to 0.368 | 0.0059 to 0.368 |
| total estimate from GNOM | 0.67 | 0.69 | 0.67 |
| **Atomistic modelling.** |  |  |  |
| Crystal structures |  |  |  |
| q range for all modelling | 0.008–0.500 | 0.008–0.500 |  |
| PepsiSAXS (r0 fixed) |  |  |  |
| No constant subtraction |  |  |  |
| χ^2^ | 15.62 | 3.3 | 6.05 |
| Predicted R_g_ (Å) | 43.11 | 43.61 | 74.07 |
| Vol (Å^3^), R0 (Å), Dro (e Å^−3^) | 17944, 1.62, 0.0007 | 208111, 1.62, 0.0027 | 242115, 1.62, 0.0023 |
| Dadimodo (https://dadimodo.synchrotron-soleil.fr/) |  |  |  |
| Starting structures | From AlphaFold2 | | |
| Rigid bodies | A: 76-170 | body1 = A: 76-172  body2 = A: 204-354, C: 1-408  body3 = A: 405-450, B: 1-226, 286-458  body4 = A: 476-546 | body1 = A: 76-172  body2 = A: 204-335, C: 1-408  body3 = A: 356-384, D: 60-136, E: 25-103  body4 = A: 405-450, B: 1-226, 286-458  body5 = A: 476-546 |
| No. of generated structures | 10 | 25 | 34 |
| χ^2^ range from PepsiSAXS | 1.49 - 2.90 | 0.65 - 1.12 | 1.09 - 1.19 |

^a^ (<https://www.synchrotron-soleil.fr/en/beamlines/swing#paragraphes_menu_left-block-7>)

**Supplementary file 1b: Yeast strains used in this study.** Strains are available on request

| **Strain number** | **Mating type** | **Genotype** | **Reference** |
| --- | --- | --- | --- |
| SL75 | h- | *ade6-704 leu1-32 ura4-D18* | Lambert et al. 2010(Lambert et al. 2010) |
| SL3456 | h+ | *pcf1-Q172A,L175A,F178A,F 179A (PIP^*^) ade6-704 leu1-32 ura4-D18* | This study |
| SL3452 | h+ | *pcf1-Y340A,W348A (ED^*^) ura4-D18 leu1-32 ade6-704* | This study |
| SL3447 | h+ | *pcf1-R147E,K150E,K154E,R161E,K168E (KER^*^) ura4-D18 leu1-32 ade6-704* | This study |
| SL2657 | h+ | *pcf1-477STOP (ΔWHD) ade6-704 leu1-32 ura4-D18* | This study |
| SL3233 | h+ | *FLAG:pcf1 ura4-D18 leu1-32 ade6-704* | This study |
| SL3647 | h+ | *FLAG-pcf1-PIP^*^ ade6-704 leu1-32 ura4-D18* | This study |
| SL3650 | h+ | *FLAG-pcf1-ED^*^ ura4-D18 leu1-32 ade6-704* | This study |
| SL3653 | h+ | *FLAG-pcf1-KER^*^ ura4-D18 leu1-32 ade6-704* | This study |
| SL3656 | h-smt0 | *FLAG-pcf1-ΔWHD ade6-704 leu1-32 ura4-D18* | This study |
| DD122 | h+ | *pcf2:GFP:NATMX ura4-D18 leu1-32 ade6-704* | This study |
| SL3727 | h+ | *pcf2:GFP:NATMX pcf1-PIP^*^ ura4-D18 leu1-32 ade6-704* | This study |
| SL3721 | h+ | *pcf2:GFP:NATMX pcf1-ED^*^ ura4-D18 leu1-32 ade6-704* | This study |
| SL3724 | h+ | *pcf2:GFP:NATMX pcf1-KER^*^ ura4-D18 leu1-32 ade6-704* | This study |
| SL3728 | h+ | *pcf2:GFP:NATMX pcf1-ΔWHD ura4-D18 leu1-32 ade6-704* | This study |
| SL3792 | h+ | *pcf2:GFP:NATMX cut11:mCherry:HYGMX ura4-D18 leu1-32 ade6-704* | This study |
| SL3602 | h+ | *rad52:GFP:KANMX pcf1::ura4^+^ ade6-704 leu1-32 ura4-D18* | This study |
| SL3611 | h+ | *rad52:GFP:KANMX pcf1-PIP^*^ ade6-704 leu1-32 ura4-D18* | This study |
| SL3609 | h+ | *rad52:GFP:KANMX pcf1-ED^*^ ade6-704 leu1-32 ura4-D18* | This study |
| SL3607 | h+ | *rad52:GFP:KANMX pcf1-KER^*^ ade6-704 leu1-32 ura4-D18* | This study |
| SL3604 | h+ | *rad52:GFP:KANMX pcf1-ΔWHD ade6-704 leu1-32 ura4-D18* | This study |
| SL3587 | h+ | *leu1-32 ade6-704 ura4-DS/E otrR::ura4^+^* | This study |
| SL3589 | h+ | *pcf1::KANMX leu1-32 ade6-704 ura4-DS/E otrR::ura4^+^* | This study |
| SL3612 | h+ | *pcf1-PIP^*^ leu1-32 ade6-704 ura4-DS/E otrR::ura4^+^* | This study |
| SL3616 | h+ | *pcf1-ED^*^ leu1-32 ade6-704 ura4-DS/E otrR::ura4^+^* | This study |
| SL3624 | h+ | *pcf1-KER^*^ leu1-32 ade6-704 ura4-DS/E otrR::ura4^+^* | This study |
| SL3596 | h+ | *pcf1-ΔWHD leu1-32 ade6-704 ura4-DS/E otrR::ura4^+^* | This study |
| VP465 | h- | *hip1::KANMX ade6-704 leu1-32 ura4-D18* | This study |

Lambert, S., K. Mizuno, J. Blaisonneau, S. Martineau, R. Chanet, K. Freon, J. M. Murray, A. M. Carr and G. Baldacci (2010). "Homologous recombination restarts blocked replication forks at the expense of genome rearrangements by template exchange." Mol Cell **39**(3): 346-359.10.1016/j.molcel.2010.07.015, PMID: 20705238
